# Impact of image preprocessing methods on reproducibility of radiomic features in multimodal magnetic resonance imaging in glioblastoma

**DOI:** 10.1002/acm2.12795

**Published:** 2019-12-27

**Authors:** Hajar Moradmand, Seyed Mahmoud Reza Aghamiri, Reza Ghaderi

**Affiliations:** ^1^ Medical Radiation Enginearing Shahid Beheshti University Tehran Iran; ^2^ Eletrical Engineering Shahid Beheshti University Tehran Iran

**Keywords:** glioblastoma, imge preprocessing, multimodal magnetic resonance imaging (mMRI), radiomics

## Abstract

To investigate the effect of image preprocessing, in respect to intensity inhomogeneity correction and noise filtering, on the robustness and reproducibility of the radiomics features extracted from the Glioblastoma (GBM) tumor in multimodal MR images (mMRI). In this study, for each patient 1461 radiomics features were extracted from GBM subregions (i.e., edema, necrosis, enhancement, and tumor) of mMRI (i.e., FLAIR, T1, T1C, and T2) volumes for five preprocessing combinations (in total 116 880 radiomics features). The robustness and reproducibility of the radiomics features were assessed under four comparisons: (a) Baseline versus modified bias field; (b) Baseline versus modified bias field followed by noise filtering; (c) Baseline versus modified noise, and (d) Baseline versus modified noise followed bias field correction. The concordance correlation coefficient (CCC), dynamic range (DR), and interclass correlation coefficient (ICC) were used as metrics. Shape features and subsequently, local binary pattern (LBP) filtered images were highly stable and reproducible against bias field correction and noise filtering in all measurements. In all MRI modalities, necrosis regions (NC: n ® ~449/1461, 30%) had the highest number of highly robust features, with CCC and DR >= 0.9, in comparison with edema (ED: n ® ~296/1461, 20%), enhanced (EN: n ® ~ 281/1461, 19%) and active‐tumor regions (TM: n ® ~254/1461, 17%). The necrosis regions (NC: $\bar{n}$ ~ 449/1461, 30%) had a higher number of highly robust features (CCC and DR >= 0.9) than edema (ED: $\bar{n}$ ~ 296/1461, 20%), enhanced (EN: $\bar{n}$ ~ 281/1461, 19%) and active‐tumor (TM: $\bar{n}$ ~ 254/1461, 17%) regions across all modalities. Furthermore, our results identified that the percentage of high reproducible features with ICC >= 0.9 after bias field correction (23.2%), and bias field correction followed by noise filtering (22.4%) were higher in contrast with noise smoothing and also noise smoothing follow by bias correction. These preliminary findings imply that preprocessing sequences can also have a significant impact on the robustness and reproducibility of mMRI‐based radiomics features and identification of generalizable and consistent preprocessing algorithms is a pivotal step before imposing radiomics biomarkers into the clinic for GBM patients.

## 1 INTRODUCTION

Glioblastoma (GBM) is the most aggressive malignant type of brain tumor, commonly occurs (de novo). Regardless of the progressions in treatment, the prognosis of GBM remains poor (median overall survival is 14 months) mainly due to the fact that GBM is remarkably heterogeneous over time and across patients.^1^

Nowadays, there is arisen interest to characterize tumor heterogeneous and phenotypes based on the high‐throughput quantified features extracted from the clinical standard of care image for providing image‐based biomarkers relating to the pathologic, genomic, proteomic, and clinical data, which is wellknown as radiomics.^2^

It is expected that radiomics takes an essential role in the current clinical oncology workflow, given that can be acquired noninvasively, and with no extra cost at any time of the treatment procedure.^3^ However, the main issue and challenging for the clinical applicability of the radiomics is the reliability and repeatability of the radiomics features^4^ across multi‐centers. Radiomics features, reliability and reproducibility can be affected by various aspects of radiomics processing (e.g., image acquisition parameters and protocols, image preprocessing algorithms, tumor segmentation, and software used for processing and feature extractions). Major of radiomics studies by concerning a different aspect of radiomics reproducibility and repeatability issue was done in computed tomography (CT) and PET modalities for limited cancer types,^5^, ^6^ and a few studies have been reported in MRI.^7^

Magnetic resonance imaging (MRI) is generally used for standard clinical care of GBM patients (i.e., Diagnoses, monitor tumor progression, and treatment response assessment). Given that MRI undergoes of various inherent acquisition artifacts and noises such as lack of standard intensity for inter‐ and intra‐scanner variability even for the same protocol, body region, and patient; intensity non‐uniformity as a result of reduced radio frequency, coil uniformity, nonlinear fields, gradient field, magnetic field, and etc.; image preprocessing method suchlike intensity normalization, bias field correction, and noise smoothing can facilitate quantitative MRI analysis and make the radiomics results more repeatable and comparable.^8^, ^9^ Currently, many attentions of the GBM mMRI‐based radiomics studies were drowned to prognosis and prediction model, while they have not used a pre‐specific image preprocessing pipeline. For an instant, in a mMRI radiomics study^10^ co‐registration, resampling (1mm^3^), and histogram intensity normalization, in other work^11^ registration, skull stripping, bias field correction, and intensity normalization, and in Ref. [12] skull stripping, registration, bias field correction and histogram matching, were implemented in mMRI preprocessing steps. Also, in Ref. [13] noise reduction, bias field correction, skull stripping, rigid registration, and intensity normalization, in another study^14^ co‐registration, noise smoothing, bias field correction, and skull stripping, in Ref. [15] co‐registration, resampling (1 mm^3^), skull stripping, noise smoothing, and intensity normalization, was used as preprocessing steps in their mMRI GBM radiomics researches. MRI preprocessing can have a considerable impact on the whole radiomics analysis.

**Figure 1:**
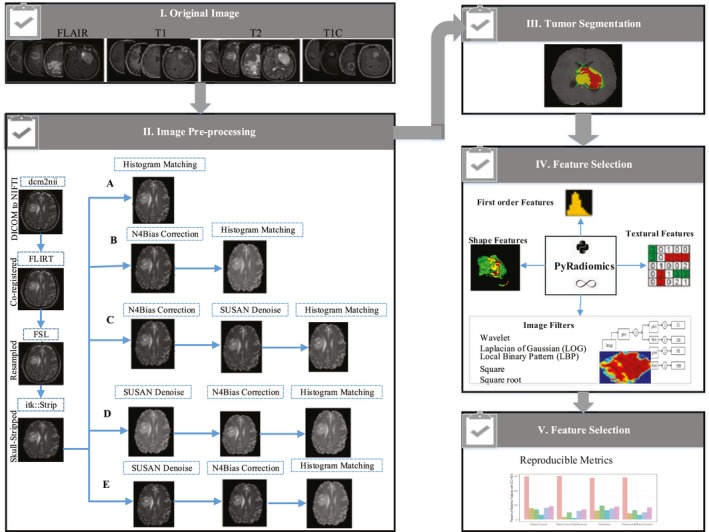
The workflow of radiomics study. Multimodal magnetic resonance images (FLAIR, T1, T2, and T1C) were subjected to several preprocessing pipelines (A, B, C, D, and E). Glioblastoma (GBM) tumor was segmented. Feature extraction was performed on multi‐regional GBM tumor by Pyradiomics. The reproducible radiomics features were selected based on reproducible metrics. FLAIR = fluid‐attenuated inversion recovery, T1C = post contrast T1 weighted

In this study, the effect of various MRI preprocessing sequences was investigated on the reliability and reproducibility of the radiomics features extracted from multi‐regional GBM tumor (i.e., Edema, Enhancement, Necrosis, and Tumor) of mMRI (FLAIR, T1, T2‐weighted (T2), and T1C), by focusing on intensity inhomogeneity correction and noise smoothing. To this end five different preprocessing cohorts were performed, and four comparisons were assessed, including (a) Baseline versus modified bias field, (b) Baseline versus modified bias field followed by noise smoothing, (c) Baseline versus modified noise reduction, and (d) Baseline versus modified noise followed bias field correction. The effect of bias field correction and noise filtering on the reproducibility of each extracted radiomics feature were quantified using concordance correlation coefficient (CCC), dynamic range (DR), and interclass correlation coefficient (ICC). The schematic of our workflow was depicted in Fig. 1. Experimental results have demonstrated that various preprocessing and sequences have affected the reproducibility and robustness of radiomics features, and identify a generalizable preprocessing step is crucially needed for providing clinical radiomics biomarkers. It is expected that our results can support researchers to select more reliable and repeatable MRI preprocessing sequences and radiomics features.

## 2 MATERIAL AND METHODS

### 2.1 Data collection

Data were downloaded from the Cancer Genome Atlas Glioblastoma Multiform (TCGA‐GBM)^16^ series, which is publically available in the Cancer Imaging Archive (TCIA)^17^ at Data Portal at [https://wiki.cancerimagingarchive.net/display/Public/TCGA-GBM]. The TCGA‐GBM series consisted of the pre‐operative and in some cases follow up MR images of 262 GBM patients and provided the pathological, genetic, and Clinical data of patients. These image sets collected from eight research centers with various scanners modalities, manufacturers, and acquisition protocols. All data were without a patient identifier, so not required for approval of the institutional review board.

In this research, 65GBM patients (40 males and 25 females with an average age of 60 yr) were selected from TCGA‐GBM series based on the availability of pre‐operative mMRI included fluid‐attenuated inversion recovery (FLAIR), T1‐weighted, T2‐weighted, and post‐contrast T1‐weighted (T1C) image sets and survival time. Further, information regarding the scanners and demographic data of these subsets of patients can be found in Table 1. 

**Table 1:** Demographic characteristics of 65 Glioblastoma patients and type of scanners were provided in this study from TCGA‐GBM series

TCGA ID	Ages (yr)	Sex(n)	Scanner (strength in T)
TCGA‐02	Ranges 18–74 Mean 54.3	Male: 10 Female 9	GE: Genesis Signa, Signa Excite
TCGA‐06	Range 40–84 Mean 62.47	Male 14 Female 6	GE (1.5, 3): Genesis Signa, Signa Excite
TCGA‐08	Range 30–76 Mean 61.8	Male 6 Female 3	GE (1.5, 3): Genesis Signa, Signa Excite
TCGA‐12	Range 46–75 Mean 64.6	Male 4 Female 2	GE (1.5): Genesis Signa, Signa HDx,Signa ExciteSiemens (1.5, 3): Avanto, Trio, Symphony
TCGA‐14	Range 59 Mean 59	Male 1 Female 0	Philips (1.5): InteraSiemens (1.5, 3): Avanto, Trio
TCGA‐19	Range 51–74 Mean 63.7	Male 2 Female 2	Siemens (1.5, 3): Avanto, Symphony, Verio
TCGA‐76	Range 50–66 Mean 59.7	Male 3 Female 3	Philips (1.5, 3): AchievaSiemens (1.5): Magnetom Vision

### 2.2 Preprocessing

Preprocessing is a series of the transformation applied to an initial image for improving the image quality and making statistical analysis more repeatable and comparable. However, for the brain MRI, there is not any pre‐specified analytic format for preprocessing and depends on the condition may diversify.

**Figure 2:**
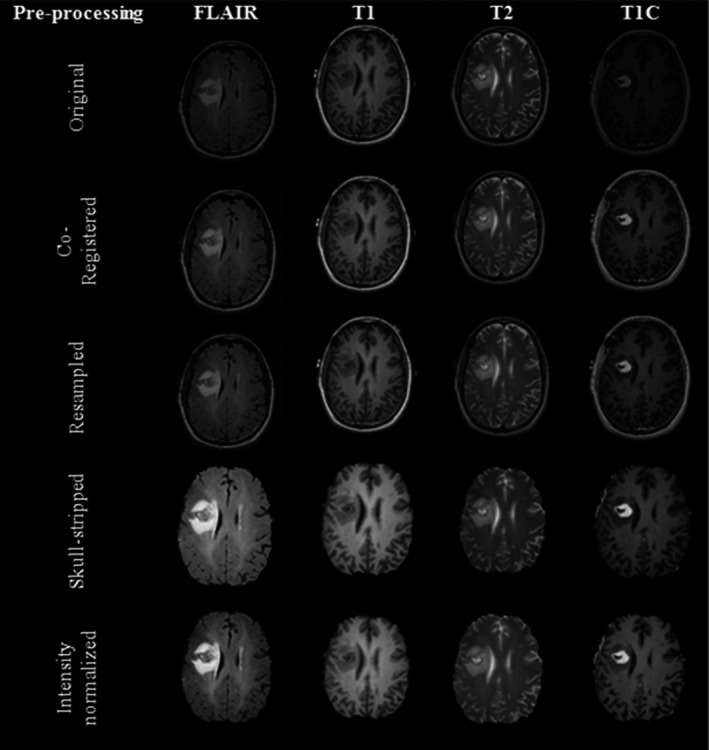
An example of our baseline preprocessing steps on a single slice of multimodal magnetic resonance imaging (MRI) (FLAIR, T1, T2, T1C) glioblastoma patient. FLAIR = fluid‐attenuated inversion recovery, T1C = Post contrast T1 weighted

In this investigation, co‐registration, resampling, skull stripping, and intensity normalization was applied as the baseline preprocessing pipeline, based on the crucial role of these methods in facilitating the radiomics analysis and accurate quantitative comparison across multi‐modal MRI volumes.^18^ An example of our baseline preprocessing pipelines on mMRI of a GBM patient was shown in Fig. 2.

Co‐registration is the process of the mapping between two images to the reference coordinate system. Resampling^19^ is commonly performed to standardize the voxel size of the database with a unique voxel resolution (e.g., 1 mm^3^) and to correct for the differences of the scanner, pixel size, and slice thickness within single or multi‐center cohort studies. Skull stripping^20^ is accomplished to remove extra cerebral tissue from brain volume and increase speed and precision of subsequent MRI processing and establishing a robust normalized result.

Variation in the brain MRI intensity distribution^21^ because of the difference in inter‐ and intra‐scanners sensitivity and acquisition parameters leads to complicated in image quantitative analysis, even for the same protocol, tissue, patient, and scanner. Consequently, intensity normalization is essential for providing the same tissue intensity scale in brain MR images across all observations to facilitate radiomics analysis^9^ and accurate quantitative comparison between MRI volumes as is expressed by Eq. (1), $g_{R}\;and\;g_{I}$ are reference image and the original image intensity respectively, $L_{R}$ and $H_{R}$ are respectively the low and high reference image intensity range.

(1) $$g_{R}=\frac{Q}{\left|g_{I}\right|}\int_{l_{R}}^{h_{R}}H_{g,I}\left(q\right)dq$$

Image preprocessing may also apply to correct other important inherent MRI acquisition artifacts such as intensity non‐uniformity and Gaussian noise. Intensity non‐uniformity, or intensity inhomogeneity, or bias of magnetic field manifests as a low‐frequency signal on MRI as a result of various causes such as fluctuation of the magnetic field.^22^ This artifact can cause variation in the intensities of the cerebral tissue regions such as gray matter, white matter, or cerebral‐spinal fluid in the different location within the brain MRI volume.

Noise is presented as high‐frequency, intensity variations in the image. Noise filtering^23^ is commonly conducted to increase the signal‐to‐noise ratio (SNR) while preserving the high‐spatial‐frequency information of the underlying image.

Various preprocessing pipelines were provided in different volumes to investigate the interplay between image preprocessing sequences, concerning intensity inhomogeneity correction and noise filtering, for mMRI on reproducibility and reliability of radiomics features. All original MRI volumes were converted to NIFTI format by using MRIcron through the dcm2nii and then were subjected to the following preprocessing pipelines.

- Co‐registration, resampling, skull stripping, and intensity normalization (baseline preprocessing pipeline).
- Co‐registration, resampling, skull stripping, bias field correction, and intensity normalization.
- Co‐registration, resampling, skull stripping, bias field correction, noise reduction, and intensity normalization.
- Co‐registration, resampling, skull stripping, noise reduction, and intensity normalization.
- Co‐registration, resampling, skull stripping, noise reduction, bias field correction, and intensity normalization.

Co‐registration was performed by using T1 mapping^24^ from SRI24 atlas^25^ image as the reference image for matching each mMRI (FLAIR, T1, T2, and T1C) volume. All images were resampled to 1 mm × 1 mm × 1 mm isotropic voxels. Skull stripping was done based on the ITK filter; the reference atlas and its relevant reference brain mask were specified by SRI24 atlas.^25^ ITK histogram matching filter^26^ was implemented to adjust the intensity scales among the patients studied to the corresponding high‐quality modality of a patient as a reference image. SUSAN (Smallest Univalue Segment Assimilating Nucleus), the low‐level image processing, was used for noise filtering. The rule of SUSAN noise filters is simple,^27^ which only takes an average over the values of contiguous pixels in uniform intensity regions is presented in Eq. (2).

(2) $$S\left(a,b\right)=\frac{\sum_{\left(i,j\right)\neq \left(0,0\right)}I\left(a+i,b+j\right)e^{-\frac{{\left(\sqrt{\left(i^{2}+j^{2}\right)}\right)}^{2}}{2\sigma^{2}}-\frac{{\left(I\left(a+i,b+j\right)-I\left(a,b\right)\right)}^{2}}{\sigma^{2}}}}{\sum_{\left(i,j\right)\neq \left(0,0\right)}e^{-\frac{{\left(\sqrt{\left(i^{2}+j^{2}\right)}\right)}^{2}}{2\sigma^{2}}-\frac{{\left(I\left(a+i,b+j\right)-I\left(a,b\right)\right)}^{2}}{\sigma^{2}}}}$$

N4ITK,^28^ a well‐known intensity inhomogeneity correction, was to correct intensity non‐uniformity. The model of image structure in this bias correction method in MR is considered to be the form of Eq. (3), where C is the corrupted image, I is the uncorrupted image, B is bias field, and n is the Gaussian noise.

(3) $$C\left(x\right)=I\left(x\right)B\left(x\right)+n\left(x\right)$$

The image model can become, $\hat{C}=\hat{I}+\hat{B}$ by taking the logarithm ($\hat{I}=\log I$) of both sides of the Eq. (3). The proposed scheme for N4ITK is developed of the original N3 (non‐parametric non‐uniformity normalization) algorithm. In this model, B‐spline fitting is improved, and the iterative component of the algorithm is optimized as described by Eq. (4), $S^{*}\left\{0\right\}$ is a modified B‐spline estimator, and ${\hat{B}}_{r}^{n}$ is the measured remaining bias field at the nth reiteration.

(4) $${\hat{I}}^{n}={\hat{I}}^{n-1}-{\hat{B}}_{r}^{n}={\hat{I}}^{n-1}-S^{*}\left\{{\hat{I}}^{n-1}-E[{\hat{I}}^{n}|{\hat{I}}^{n-1}]\right\}$$

FMRIB Software Library (FSL)^29^ was used for co‐registration (FLIRT)^30^ as well as for resampling. Skull‐stripping and de‐noising approach (SUSAN) was applied by Cancer Imaging Phenomics Toolkit (CaPTK).^31^ FSL and CaPTK are publicly available through the ://fsl.fmrib.ox.ac.uk, and ://www.nitrc.org/projects/captk, respectively. N4 bias field correction and histogram matching filter were performed respectively by the N4ITK and ITK (Histogram Matching Image Filter) with the 3D slicer version 4.8.1.

### 2.3 Tumor segmentation

In radiomics studies, tumor segmentation is also a common source of variation in quantitative image analyzing because of intra‐ and inter‐reader and software variability. To minimize the effects of tumor segmentation on our radiomics reproducibility analyze, the results of previously published work,^15^ publically accessible through TCIA, were used for tumor segmentation labels. GLISTR boost, the modified version of GLISTR (GLioma Image SegmenTation and Registration) software, was applied for tumor segmentation based on a hybrid generative‐discriminative model.

To segment the brain images into normal and tumor tissue labels, concomitant registration, the atlas of normal brain scans to brain scans with GBM tumors, and segmentation approaches based on an Expectation‐Maximization (EM) framework using the tumor growth model were applied (a generative part, i.e., GLISTR).

To provide more reliable and accurate tumor labels, gradient boosting multi‐class classification was implemented according to the information of the multi patients (a discriminating part). Tumor segmentation was completed and further modified by establishing a probabilistic Bayesian approach based on the statistical intensity of the patient‐specific from the multi‐MRI modalities. In comparison with GLSTR, which relies on a single tissue‐seed point for each brain label, GLISTR boost can more accurately segment tumor, by model the intensity distribution of multiple seed points for each brain tissue label.

Three tumor labels were outlined include edema, enhancement, and necrosis subregions. The edema region is described as a hyper‐intensity signal on FLAIR and T2 images. The enhancement area biologically occurs because of blood‐brain barrier disruption leading leakage of contrast and can be identified as the high‐intensity part of the tumor in T1C in comparison with T1voume. The necrosis region is a hypo‐intensity precinct of the tumor core in T1C, as necrotic cells do not react with the contrast agent. Tumor region comprised of enhancement and necrosis area.^32^ Here, MATLAB (MATLAB 2017b, commercial software package) was applied on each manual correction segmented label of each patient, in order to extract tumor labels.

### 2.4 Feature extraction

Radiomics features categorize into four classes, including the first order features, shape features, texture features, and image filtered feature. Texture features were implemented in this research include gray level co‐occurrence matrix (GLCM),^33^ gray level run length matrix (GLRLM),^34^ gray level size zone matrix (GLSZM),^35^ neighboring gray tone difference matrix (NGTDM),^36^ and gray level dependence matrix (GLDM).^37^ Image filtered types applied were wavelet transformation, Laplacian of Gaussian (LoG), square, square root, and local binary pattern (LBP). First order and texture features were obtained from the original image and also filtered images. Shape features as an indicator of ROI (region of interest) morphology were only extracted from original images.

Wavelet transform^38^ is a powerful tool for multiscale feature extraction, represents images hierarchically based on a scale (s) and resolution (b) as is given in Eq. (5).

(5) $$Wf\left(s,c\right)=\frac{1}{\sqrt{s}}\int_{-\infty }^{\infty }\psi \left(\frac{t-c}{s}\right)f\left(t\right)dt$$

It analyzes high‐spatial frequency phenomena localized in space, thus it can effectively extract information derived from localized high‐frequency signals. Wavelet transform was calculated per eight decomposition levels applying either a high pass filter or a low pass filter in the original volumes.

Laplacian of Gaussian (LoG)^39^ filter is the process of two steps, firstly smooth image by using a Gaussian filter, then applying the Laplacian to find edges (areas of gray level rapid change) as is calculated by Eq. (6), (a, b) is the spatial coordinates of the image pixel, and σ is the kernel size of the Gaussian filter. In this study, the LoG filter was implemented by two kernel size values (σ = 1, and 2 mm).

(6) $$LoG\left(a,b\right)=-\frac{1}{\pi \sigma^{4}}\left[1-\frac{a^{2}+b^{2}}{2\sigma^{2}}\right]e^{-\frac{a^{2}+b^{2}}{2\sigma^{2}}}$$

Square and square root image filter are tagged as Gamma modifiers. The square filter is accomplished by taking the square of image intensities ($g_{I}$) is given by Eq. (7), and the square root filter by taking the square root of the absolute value of image intensities is presented in Eq. (8), then the results are modified into the original image intensity range.

(7) $$Square\left(g_{I}\right)={\left(\frac{g_{I}}{\sqrt{max\left|g_{I}\right|}}\right)}^{2}$$

(8) $$Squareroot\left(g_{I}\right)=\left\{\begin{array}{cc} \sqrt{max\left|g_{I}\right|g_{I}} & g_{I}>0\\ -\sqrt{-max\left|g_{I}\right|g_{I}} & g_{I}<0 \end{array}\right.$$

Local Binary Pattern (LBP)^40^ relies on labeling a binary value to each pixel of the image by thresholding the neighboring pixels based on the central pixel value, the histogram of these labels considers as texture features.

LBP can be represented in the form of Eq. (9), P denotes the surrounding pixel sampling in the circle neighbors of the radius r, and $2^{p}$ various binary patterns are generated is corresponding to the size of the histogram. The $f\left(i_{p}-i_{c}\right)$, is defined as presented in Eq. (10), $i_{c}$ is the intensity of the central pixel, $i_{p}$ is the intensity of the neighboring pixel.

(9) $$LBP\left(P,r\right)=\sum_{p=0}^{p-1}f\left(i_{p}-i_{c}\right)2^{p}$$

(10) $$f\left(i_{p}-i_{c}\right)=\left\{\begin{array}{cc} 1 & ifi_{p}\geq i_{c}\\ 0 & fi_{p}<\;i_{c} \end{array}\right.$$

The radiomics features classes implemented in this study can be found in Table 2. PyRadiomics, as a standard open‐source Python package, was employed for implementing a streamlined and reproducible standard tested platform for the radiomics features extraction task.

**Table 2:** Different radiomics features classes analyzed in this study. GLCM = gray‐level co‐occurrence matrix, GLDM = gray level dependence matrix, GLRLM = gray‐level run‐length matrix, GLSZM = gray‐level size‐zone matrix, NGTDM = neighboring gray tone difference matrix

Category	Radiomic features
Shape features	1‐Elongation, 2‐ Flatness, 3‐ LeastAxisLength, 4‐ MajorAxisLength, 5‐ Maximum2DDiameterColumn, 6‐ Maximum2DDiameterRow, 7‐ Maximum2DDiameterSlice, 8‐ Maximum3DDiameter,9‐ MeshVolume, 10‐MinorAxisLength, 11‐SurfaceArea, 12‐ VoxelVolume,13‐ SurfaceVolumeRatio, 14‐ Sphericity
First order	1‐Energy ,2‐ Entropy, 3‐ InterquartileRange, 4‐ Kurtosis, 5‐ Maximum,6‐ MeanAbsoluteDeviation, 7‐Mean 8‐ Median, 9‐ Minimum, 10‐ RobustMeanAbsoluteDeviation, 11‐ RootMeanSquared, 12‐ Skewness, 13‐ TotalEnergy, 14‐ Uniformity, 15‐ Variance, 16‐ 10Percentile, 17‐ 90Percentile, 18‐Range
GLCM	1‐Autocorrelation, 2‐ClusterProminence, 3‐ClusterShade, 4‐ClusterTendency, 5‐Contrast, 6‐Correlation, 7‐DifferenceAverage, 8‐DifferenceEntropy, 9‐DifferenceVariance, 10‐Id, 11‐Idm, 12‐Idmn, 13‐dn, 14‐Imc1, 15‐Imc2, 16‐InverseVariance,17‐JointAverage, 18‐JointEnergy, 19‐JointEntropy, 20‐MCC, 21‐MaximumProbability, 22‐SumAverage, 23‐SumEntropy, 24‐SumSquares
GLRLM	1‐GrayLevelNonUniformity, 2‐GrayLevelNonUniformityNormalized, 3‐GrayLevelVariance, 4‐HighGrayLevelRunEmphasis, 5‐LongRunEmphasis, 6‐LongRunHighGrayLevelEmphasis, 7‐LongRunLowGrayLevelEmphasis, 8‐LowGrayLevelRunEmphasis, 9‐RunEntropy, 10‐RunLengthNonUniformity, 11‐RunLengthNonUniformityNormalized, 12‐RunPercentage, 13‐RunVariance, 14‐ShortRunEmphasis, 15‐ShortRunHighGrayLevelEmphasis, 16‐ShortRunLowGrayLevelEmphasis
GLSZM	1‐GrayLevelNonUniformity, 2‐GrayLevelNonUniformityNormalized, 3‐GrayLevelVariance, 4‐HighGrayLevelZoneEmphasis, 5‐LargeAreaEmphasis, 6‐LargeAreaHighGrayLevelEmphasis, 7‐LargeAreaLowGrayLevelEmphasis, 8‐LowGrayLevelZoneEmphasis, 9‐SizeZoneNonUniformity, 10‐SizeZoneNonUniformityNormalized ,11‐SmallAreaEmphasis, 12‐SmallAreaHighGrayLevelEmphasis, 13‐SmallAreaLowGrayLevelEmphasis, 14‐ZoneEntropy, 15‐ZonePercentage, 16‐ZoneVariance
NGTDM	1‐Busyness, 2‐Coarseness, 3‐Complexity, 4‐Contrast, 5‐Strength
GLDM	1‐DependenceEntropy, 2‐DependenceNonUniformity, 3‐DependenceNonUniformityNormalized, 4‐DependenceVariance, 5‐GrayLevelNonUniformity, 6‐GrayLevelVariance, 7‐HighGrayLevelEmphasis, 8‐LargeDependenceEmphasis, 9‐LargeDependenceHighGrayLevelEmphasis, 10‐LargeDependenceLowGrayLevelEmphasis,11‐LowGrayLevelEmphasis,12‐SmallDependenceEmphasis, 13‐SmallDependenceHighGrayLevelEmphasis,14‐SmallDependenceLowGrayLevelEmphasis

### 2.5 Statistical analysis

In this study, the concordance correlation coefficient (CCC) and the intraclass correlation coefficient (ICC), the most popular indices for assessing agreement between continuous variables in reproducibility studies, alongside the dynamic range (DR) were employed to evaluate the stability and reproducibility of the radiomics features.

The Pearson correlation coefficient is usually used for assessing the linear correlation between pairs of quantitative variables, however, it is inappropriate for agreement measuring. Agreement and correlation indicate the different concepts of relationship. An excellent correlation between two variables does not support that these two observers can produce the same outcome insomuch correlation fails in identifying the systematic variations between observers, whereas agreement regarded whether various mechanisms, raters or methods, produce identical results of the calculated responses.

The CCC calculated as described with 95% confidence intervals to test the agreement between raters^41^ by Eq. (11), μ is a mean value, and σ refers to the standard deviation, related to this work it was between the respective values of the baseline and modified preprocessed images.

(11) $$CCC\left(b,m\right)=1-\frac{{\left(\mu_{b}-\mu_{m}\right)}^{2}+\sigma_{b}^{2}+\sigma_{m}^{2}-2\delta_{b}\delta_{m}}{{\left(\mu_{b}-\mu_{m}\right)}^{2}+\sigma_{b}^{2}\sigma_{m}^{2}}$$

Besides, for assaying inter‐rater reliability intraclass correlation coefficient (ICC) is an appropriate index,^42^ which evaluates both sides of the correlation and agreement among raters, is given by Eq. (12).

(12) $$ICC\left(1,1\right)=\frac{\sigma_{b}^{2}}{\sigma_{b}^{2}+\sigma_{e}^{2}}=\frac{\frac{MS_{B}-MS_{W}}{K}}{\frac{MS_{B}-MS_{W}}{K}+MS_{W}}$$

Albeit the ICC and CCC values are equivalent or similar in particular cases, they differ in two important respects; (a) The ICC is defined for fixed or random observers, whereas the CCC is usually proposed for fixed observers; and (b) The ICC indexes are required in the assumption of the ANOVA model, different versions of ICCs can give different results depending on the chosen ANOVA models, in contrast, the CCC is assessed without assuming the ANOVA model.

The entire physiological range of feature values observes across patients can also impact the reliability and stability of the features. The dynamic range (DR)^43^ is used as an explanation of the physiological characteristics to determine the reliability and informative radiomic features. The DR is calculated for the baseline and modified sample population with the Eq. (13), b denotes the baseline group, m indicates the modified collection, n is the number of patients, and j is the case index for the ith features.

(13) $$DR_{i}\left(b,m\right)=1-\frac{1}{n}n\sum_{j=1}^{}\frac{\left|b{\left(j\right)}_{i}-m{\left(j\right)}_{i}\right|}{\left(Max(range{\left(b,m\right)}_{\text{i}}\right)-Min\left(range{\left(b,m\right)}_{i}\right)}$$

The range of DR is between 0 and 1, and features with the DR values closer to one, have more inclination to be considered as robust features against perturbation and hold comparatively reliable information.

In this study, radiomic features with CCC and DR > 0.9 were considered as robust and reproducible features; and the Intraclass correlation coefficient (ICC) with 95% confident intervals, higher than 0.9 and between 0.75–0.9 considered as excellent and good reproducible cohorts, respectively.^44^

Statistical analysis was implemented with r software; version 3.5.1, including “epir” and “irr” packages, were used for calculating CCC and ICC respectively.

## 3 RESULTS

The effect of MRI image preprocessing including or excluding intensity inhomogeneity correction and noise filtering on the reproducibility of the radiomics features was evaluated by four comparisons as follows: (a) Baseline versus modified bias field; (b) Baseline versus modified bias field followed by noise filtering; (c) Baseline versus modified noise reduction, and (d) Baseline versus modified noise followed bias field correction.

In this study, for each patient 1,461 radiomics features were extracted from GBM sub‐regions (i.e., edema, necrosis, enhancement, and tumor) of mMRI (i.e., FLAIR, T1, T1C, and T2) volumes for five preprocessing combinations (in total 23 376 radiomics features). Statistical analysis was computed over all of the possible measurements.

In Table 3, the total numbers of radiomics features and their percentage with high robustness (CCC >= 0.9 and DR >= 0.9) across all MRI sequences for each GBM phenotype, was summarized. Overall, the total number of high robustness features was extracted from the necrosis region (NC: $\sum_{i=1}^{16}n_{i}$ = 7173; 30.6%) were higher than the number of features were extracted from edema regions (ED: $\sum_{i=1}^{16}n_{i}$ = 4730; 20.2%), enhancement regions (EN: $\sum_{i=1}^{16}n_{i}$=4490; 19.2), and active tumor regions (TM: $\sum_{i=1}^{16}n_{i}$ = 4066; 17.3%) overall cohorts of comparisons and mMRI modalities.

**Table 3:** Total number ($\sum_{i=1}^{4}n_{i}$) of features and their percentage ($\frac{\sum_{i=1}^{4}n_{i}}{4*1461}$%), where $n_{i}$ refers to the number of features with high robustness (CCC& DR >= 0.9) extracted from each GBM sub‐regions of each mMRI (FLAIR, T1, T1C, and T2) volumes. The percentages of features extracted from necrosis regions across all cohorts and MRI modalities were higher than the other phenotypes

GBM phenotype	I (Base vs modified bias field)		II (Base vs modified bias field &noise)		III (Base vs modified noise)		IV (Base vs modified noise& bias field)	
	$\sum_{i=1}^{4}n_{i}$	Percent (%)	$\sum_{i=1}^{4}n_{i}$	Percent (%)	$\sum_{i=1}^{4}n_{i}$	Percent (%)	$\sum_{i=1}^{4}n_{i}$	Percent (%)
Edema	1258	21.5	1116	19	1273	21.7	1083	18.5
Enhancement	1236	21.1	1222	20.9	977	16.7	1055	18
Necrosis	1915	32.7	1849	31.6	1734	29.6	1675	28.6
Active tumor	1012	17.3	1072	18.3	1020	17.4	962	16.4

In addition, the average number of highly reproducible features (ICC >= 0.9) for baseline comparison with modified bias field (cohort I:$\bar{n}$ ~339/1461, 23.2%), were higher than cohort II ($\bar{n}$ ~329/1461; 22.5%), cohort III ($\bar{n}$ ~313/1461, 21.4%), and cohort IV ($\bar{n}$ ~298/1461; 20.4%). In Table 4., the number of radiomic features extracted from FLAIR images with ICC greater than 0.9 (excellent group), and between 0.75 and 0.9 (good cohort), for all measurements were presented.

**Table 4:** The total number ($\sum_{i=1}^{4}f_{i}$) of features and their percentage ($\frac{\sum_{i=1}^{4}f_{i}}{n\times 4}$%), where $f_{i}$ reflect the number of features with ICC >= 0.9 (excellent) or 0.75 <= ICC < 0.9 (good) extracted from multi‐regions of GBM (edema, enhance, necrosis, and active tumor) in FLAIR modality, and n, refer to the number of each feature category. ICC = Intraclass correlation coefficient, LBP = local binary pattern, LoG = Laplacian of Gaussian

Image type	Feature category (n)	ICC	I (Base vs modified bias field)		II (Base vs modified Bias field &noise)		III (Base vs modified noise)		IV (Base vs modified noise & bias field)	
			$\sum_{i=1}^{4}f_{i}$	Percent (%)	$\sum_{i=1}^{4}f_{i}$	Percent (%)	$\sum_{i=1}^{4}f_{i}$	Percent (%)	$\sum_{i=1}^{4}f_{i}$	Percent (%)
Original	Shape (14)	ICC>=0.9	56	100	56	100	56	100	56	100
		0.75<=ICC < 0.9	0	0	0	0	0	0	0	0
	First order (18)	ICC>=0.9	5	6.9	2	2.7	9	12.5	2	2.7
		0.75<=ICC < 0.9	18	25	17	23.6	16	22.2	15	20.8
	Texture (75)	ICC>=0.9	42	14	25	8.3	57	19	37	12.3
		0.75<=ICC < 0.9	98	32.6	70	23.3	91	30.3	105	35
Wavelet	First order (144)	ICC>=0.9	60	10.4	73	12.6	51	8.8	34	5.9
		0.75<=ICC < 0.9	205	35.6	206	35.7	172	29.8	182	31.6
	Texture (600)	ICC>=0.9	501	20.8	444	18.5	303	12.6	344	14.3
		0.75<=ICC < 0.9	806	33.5	875	36.4	667	27.8	790	33
LBP	First order (30)	ICC>=0.9	58	48.3	59	49.1	51	42.5	59	49.1
		0.75<=ICC < 0.9	55	45.8	41	34.1	50	41.6	44	36.6
	Texture (208)	ICC>=0.9	573	68.8	582	70	579	69.5	571	68.6
		0.75<=ICC < 0.9	86	10.3	77	9.2	82	9.8	84	10
LoG	First order (36)	ICC>=0.9	7	4.8	6	4.1	5	3.4	7	4.8
		0.75<=ICC < 0.9	22	15.2	24	16.6	21	14.5	24	16.6
	Texture (150)	ICC>=0.9	82	13.6	49	8.1	106	17.6	73	12.1
		0.75<=ICC < 0.9	159	26.5	91	15.1	165	27.5	158	26.3
Square	First order (18)	ICC>=0.9	2	2.7	3	4.1	12	16.6	4	5.5
		0.75<=ICC < 0.9	27	37.5	27	37.5	31	43	27	37.5
	Texture (75)	ICC>=0.9	20	6.6	12	4	46	15.3	31	10.3
		0.75<=ICC < 0.9	118	39.3	98	32.6	126	42	101	33.6
Square root	First order (18)	ICC>=0.9	5	7	3	4.1	5	7	2	2.7
		0.75<=ICC < 0.9	12	16.6	11	15.2	10	13.8	11	15.2
	Texture (75)	ICC>=0.9	49	16.3	44	14.6	53	17.6	44	14.6
		0.75<=ICC < 0.9	92	30.6	71	23.6	85	28.3	89	29.6

**Figure 3:**
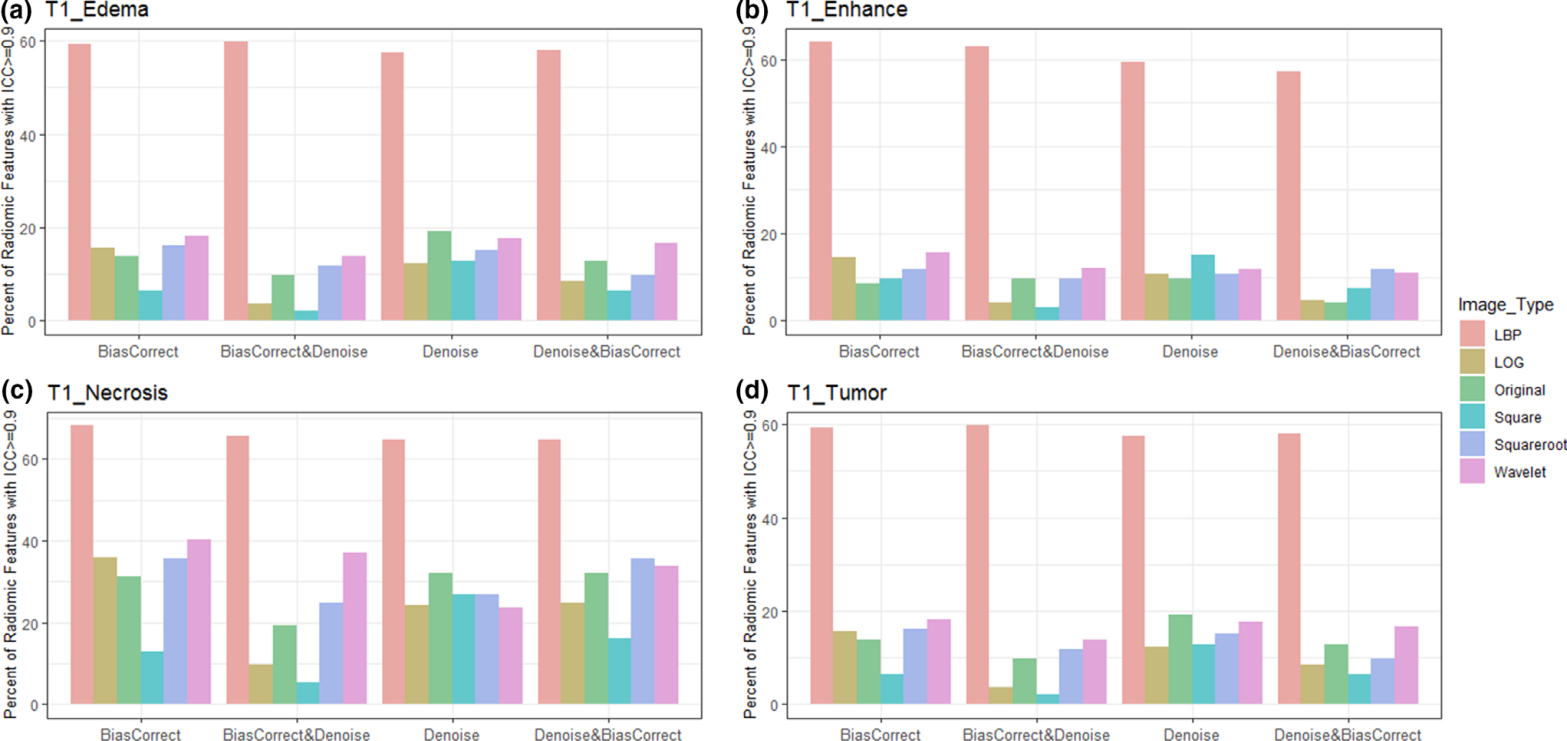
Bar plot of percent radiomics features extracted from various Glioblastoma phenotypes (a) edema, (b) enhancement, (c) necrosis, and (d) active tumor) of T1 images with ICC>= 0.9. Feature extracted from LBP filtered image and necrosis region were very reproducible. LBP = local binary pattern, LoG = Laplacian of Gaussian

**Figure 4:**
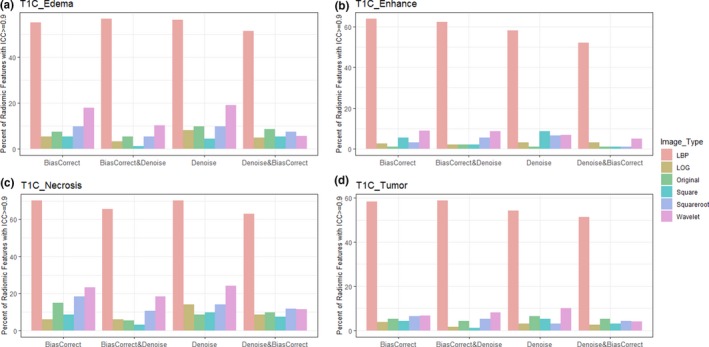
Bar plot of percent radiomics features extracted from various Glioblastoma phenotypes (a) edema, (b) enhancement, (c) necrosis, and (d) active tumor) of post contrast T1 weighted images with ICC> =0.9. Feature extracted from LBP filtered image and necrosis region were very reproducible. LBP = Local Binary Pattern, LoG = Laplacian of Gaussian

**Figure 5:**
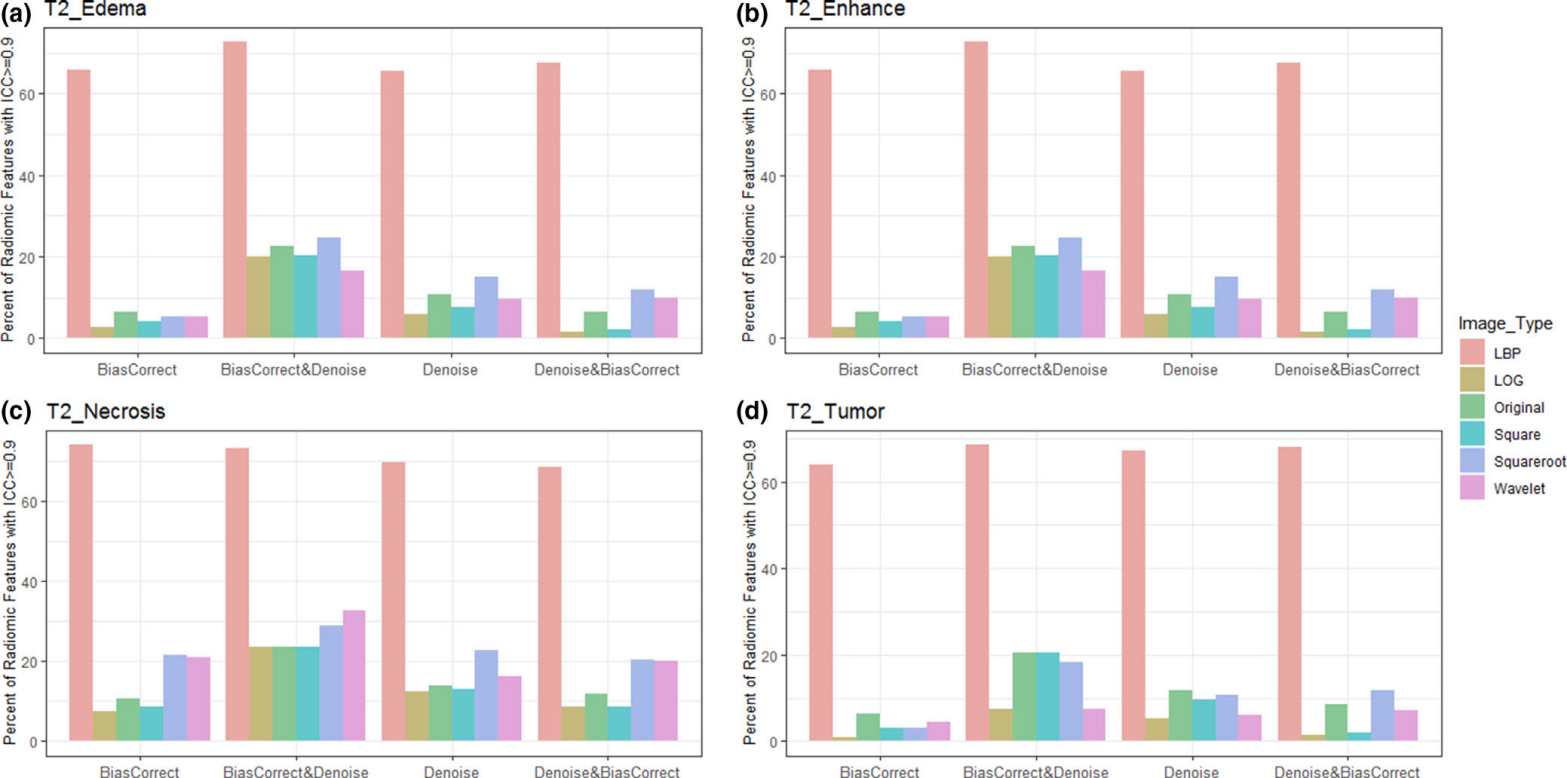
Bar plot of percent radiomics features extracted from various Glioblastoma phenotypes (a) edema, (b) enhancement, (c) necrosis, and (d) active tumor) of T2 images with ICC>= 0.9. Feature extracted from LBP filtered image and necrosis region were very reproducible. LBP = local binary pattern, LoG = Laplacian of Gaussian

Furthermore, the percentage of radiomics feature extracted from multiregional GBM in T1, T1C, and T2 sequences with excellent reproducibility (ICC >= 0.9) was shown in Figs. 3, 4, 5 respectively.

## 4 DISCUSSION

Glioblastoma (GBM) is the most aggressive malignant type of primary brain tumor. The prognosis of the GBM remains poor, mainly since it is remarkably heterogeneous over time and across patients.^1^

The promising evidence of MRI‐based radiomics, confirms that GBM radiomics can non‐invasively and cost‐effectively depict GBM tumor heterogeneity,^2^ and predict patients’ outcomes.^10^, ^11^ However, the critical issue for adoption of radiomics features in the routine clinical oncology workflow as validating biomarkers is their robustness and reproducibility.

Magnetic resonance images undergo of some intrinsic acquisition artifacts, and different scanners with various acquisition parameters, and protocols, severely impeded the multicenter applicability of MRI‐based radiomics,^8^ the image preprocessing takes an essential part in the facilitating quantification analysis and make more repeatable and comparable results. However, the lack of pre‐specific image standardization methods in the mMRI‐based radiomics analysis may also affect the reproducibility and reliability of the features.

This investigation was designed to assay the impact of various image preprocessing combinations on the robustness and reproducibility of mMRI‐based radiomics in GBM tumor. The high throughout radiomics features were extracted by using PyRadiomics a standard open‐source platform,^45^ provide more comparable and reproducible results for other research centers. We expected that our investigation would have great potential to support the researchers for selecting more stable mMRI‐based radiomics features of GBM patients.

Our results revealed that necrosis features were more stable, and less sensitive to the intensity inhomogeneities and noise, in comparison with the features derived from other GBM phenotypes. Necrosis features were used efficaciously in different radiomics studies and demonstrated that features such as necrosis pattern and necrosis volume were remarkably associated with GBM survival and mutations.^46^

Besides, our results reconfirmed that shape features are less influenced by bias field and noise artifacts. This can be easily interpreted due to shape features are not or less correlated to the gray level intensity distribution. Shape features reproducibility were predominantly reported by multiple radiomics studies, mostly in CT modality^5^ and in the limited literature for MR images.^47^, ^48^

Furthermore, the stability of LBP‐filtered was substantially higher than the other filtered (i.e., wavelet, LoG, square, and square root) volumes. In contrast, square‐filtered images were the most sensitive to the intensity inhomogeneity and had the least number of reproducible features.

Local binary pattern (LBP) is a practical texture feature with excellent image descriptor ability,^40^ it provides local‐level feature extraction to identify small gray level differences. Although there is plausible evidence of the outstanding performance of LBP filter in various aspects, it has been rarely used in radiomic studies.^6^

Moreover, the total numbers of reproducible features for bias field correction and bias filed correction followed by noise smoothing, were higher than noise reduction or noise smoothing followed by bias field correction over MRI sequences. This finding is consolidated by previous literature, which indicated that bias field correction preceded noise reduction more improving the MR image quality than noise filtering followed by bias field correction.^49^, ^50^

Even though the qualities of our findings are robust, the reproducibility and repeatability of radiomics features were investigated on only five popular preprocessing combinations. Further validation is needed to identify the optimum and more generalizable image preprocessing method for MR image standardization in GBM patients.

## 5 CONCLUSION

In conclusion, four critical issues were deduced from our findings. Firstly, radiomics features extracted from necrotic regions were the most robust in comparison with other GBM phenotypes (edema, and enhancing active tumor) independent of MRI sequences. Secondly, shape features were the most robust and reproducible in all MRI sequences and GBM phenotypes. Thirdly, the LBP‐filtered images were less sensitive to intensity inhomogeneity, and noise artifacts, and had better reproducibility features in comparison with the other image types (i.e., original, Wavelet, LoG, square, square root). Finally, if researchers are interested in modifying intensity inhomogeneity and noise simultaneously, bias field correction followed by noise filtering introduce more stable and reproducible radiomics features than noise filtering followed by bias field correction. For future work, we will explore the added value of these features, along with the prognostic model for GBM patients.

## CONFILICT OF INTEREST

There is no conflict of interest declared in this article.
